# Unique root anatomy of mandibular second premolars: clinical strategies for effective disinfection and preservation of dentine structure in root canal treatment—a case report

**DOI:** 10.3389/fdmed.2024.1403138

**Published:** 2024-05-09

**Authors:** Ji Wook Jeong, Erika Silguero Gonzalez, Scott R. Makins, Timothy Kirkpatrick

**Affiliations:** Department of Endodontics, School of Dentistry, The University of Texas Health Science Center at Houston, Houston, TX, United States

**Keywords:** CBCT, C-shape, mandibular second premolar, non-surgical root canal treatment, radicular groove, root canal anatomy

## Abstract

It is difficult to predict the outcomes of non-surgical root canal treatment (NSRCT) for mandibular second premolars with multiple root canals. In these teeth, the complicated anatomy with fin(s), or a C-shape, and possible secondary canal(s) varies unpredictably. The morphology of the root canals provides shelters for bacteria to remain, regrow, and form biofilms despite the endodontic treatment. Moreover, the prevalence of mandibular second premolars with multiple canals is extremely infrequent. Therefore, the clinical management of NSRCT in such cases is not sufficiently reported. This case report introduces two cases of NSRCT for mandibular second premolars with a radicular groove and also presents the appropriate clinical strategies and techniques. Cone-beam computed tomography (CBCT) imaging was used preoperatively. After the completion of NSRCT, CBCT was reused to review the completed procedures using the Nerve Canal tool in CS 3D Imaging v3.2.9 and v3.8.7. Five canals were obturated in case 1, and four canals in case 2 along with a C-shape morphology. Analyzing the configuration of the root canals by CBCT was critical to achieve successful NSRCT because the numbers, exits, configurations, and volumes of the secondary canals were not anticipated after reviewing 2D radiographs. Based on the interpretation, the advanced protocols of NSRCT were planned: first, augmentation of chemo-mechanical cleaning, but minimizing the loss of dentine; second, the tactile examination to locate and negotiate the orifices of the secondary canals; and lastly, the optimized plan and technique for root canal filling of the complex canal systems.

## 1 Introduction

The etiology of pulpal and related apical tissue disease is bacteria in the root canal system of the tooth (1). The purpose of non-surgical root canal treatment (NSRCT) is to remove and/or kill bacteria in the root canal systems of the tooth. Before the root canal treatment, the clinician needs to be knowledgeable about the morphology of the root canal system harboring the bacteria.

Regarding the predictability of the outcomes of NSRCT, there are three independent variables for the prognosis of treatment: anatomy of the root canal system, bacterial population, and clinical strategy and performance.

The anatomy of canals can be clinically characterized as simple or complex, depending on the accessibility for endodontic instruments. Complex root canal morphology includes canals with acute and/or multiple curves, a fin or isthmus, canals that are C-shaped or obliterated by tertiary dentine, and atypical “extra” canals. If the presence of a complex canal system is not initially recognized, and the complexity of instrumentation and obturation is underestimated, the prognosis/success rate of the treatment will be decreased (2, 3).

Biofilms protect bacteria from exposure to antibacterial treatments and also increase their resistance to antimicrobials (4). It is not possible to completely remove the biofilms within the root canal system (5). The anatomical complexity of root canals hampers the chemo-mechanical disinfection processes used in NSRCT, resulting in the formation or enhancement of biofilms after instrumentation (6). Therefore, it is important to use the advanced chemo-mechanical protocols of endodontic irrigation techniques when treating complicated root canal anatomies.

Clinical strategy and performance of NSRCT needs to vary with the canal system anatomy and the associated presence of biofilms (3). Cone-beam computed tomography (CBCT) imaging has become a powerful method to delineate the complex configuration of the root shape and morphology of the canals, as well as the appearance of bone loss in the jaws (7). In addition, CBCT is useful to verify the calcification of the canals. Therefore, it is necessary to maximize the use of CBCT images for the formulation of an appropriate clinical strategy when treating complex root canals.

The root canal morphology of mandibular second premolars is normally simple, with one canal terminating at the apex (8, 9). The anatomy of the mandibular second premolar with three to five canals and a radicular groove is extremely infrequent, only ranging up to 0.4% (8–10). In addition, the presence of fins or C-shaped configurations of the canals is commonly associated with mandibular second premolars exhibiting a radicular groove (11, 12).

This case report presents two cases of NSRCT for mandibular second premolars with multiple canals and a radicular groove. This case report aims to introduce clinical strategies and techniques designed to provide successful treatment for such cases.

## 2 Case report

This report is composed of two case reports. One case was selected from patients referred to the extramural dental practice of the University of Texas Health Science Center at Houston School of Dentistry and the other case was selected from the patient population of the institution's Advanced Education Program in Endodontics. The first case was treated by a board-certified endodontist (JJ), and an endodontic resident (EG) provided treatment for the second case under faculty supervision. Conventional periapical radiographs and CBCT imaging (CS 9000; Carestream Dental, Atlanta, GA, USA) were used for the radiographic examinations. Both NSRCTs were performed under a dental microscope. No identifiable information was included to maintain the anonymity of the patients. In addition, the patients’ medical histories and medications were not relevant to the purpose of the case reports and are not reported.

### 2.1 Case 1

**Figure 1:**
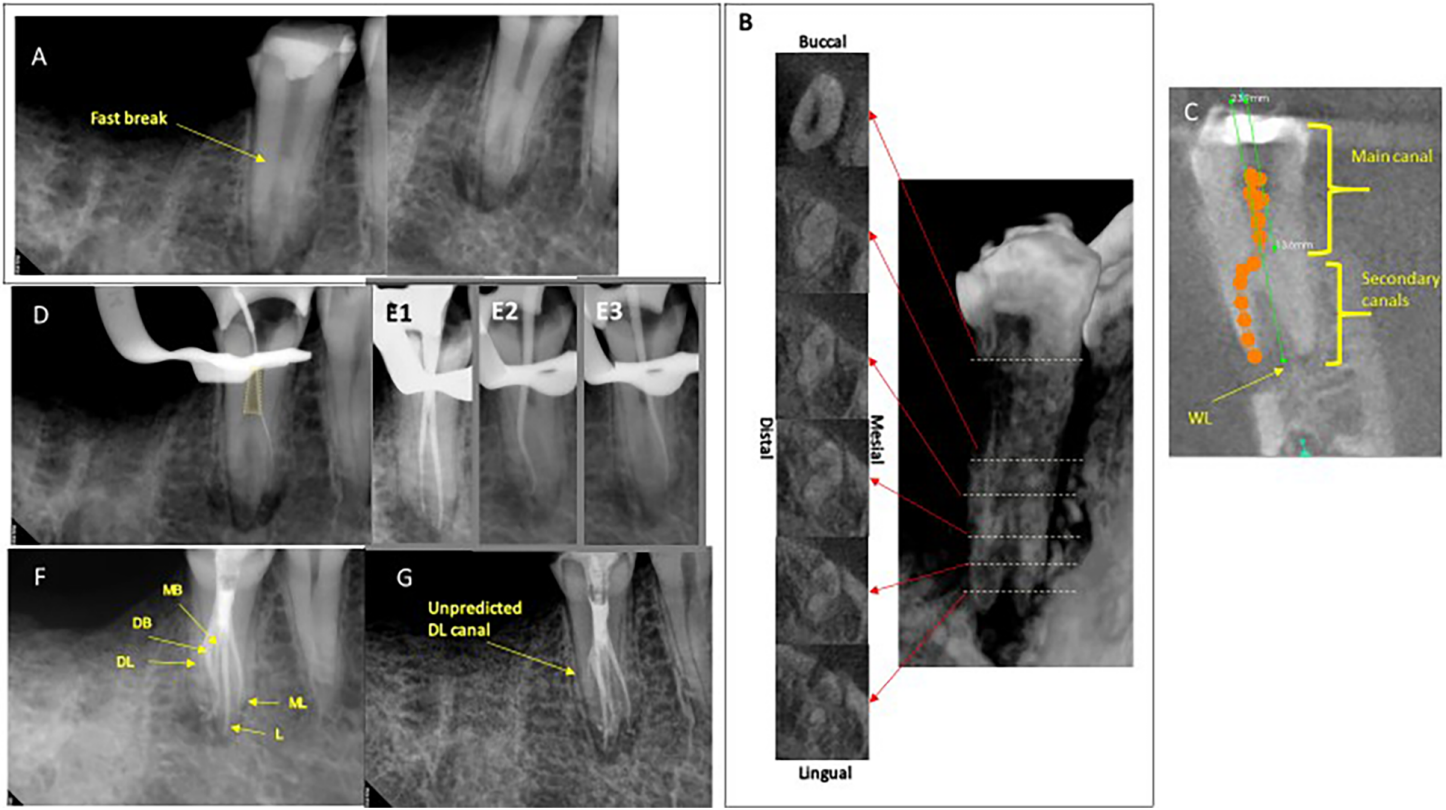
(**A**) Differently angled preoperative radiographs showing the multiple root apices of tooth 45. (**B**) CBCT scan: (Left) Note the radicular groove distally at the apical one-third of the root and it is obscure to count the numbers of the secondary canals. (Right) A snapshot of 3D construction corresponding to the serial axial views. (**C**) A CBCT scan: a coronal view showing premeasuring the length of a main canal and full lengths of the root canals. (**D**) A radiograph showing a handfile for verifying the working length. Note the method of locating the secondary canals preserving the dentin of the primary canal (yellow trapezoid). (**E**) Three radiographs showing trial gutta-percha cones. (**E1**) MB and L. (**E2**) DB. (**E3**) ML. (**F,G**) Differently angled postoperative radiographs. Note that DL was unpredictably obturated without mechanical shaping.

A 28-year-old patient was referred for endodontic treatment of tooth 45. The diagnoses were previously initiated therapy and asymptomatic apical periodontitis (Figure 1A). Because a complex configuration of the root canal system including a radicular groove was predicted, CBCT was carried out preoperatively and the anatomy of the root was analyzed (Figure 1B). One main canal and four calcified secondary canals [mesiobuccal (MB), mesiolingual (ML), distobuccal (DB), lingual (L)] were detected via the CBCT scan. Considering the complicated anatomy of the root and the canal system, an optimized clinical strategy was developed for the treatment: first, premeasuring the working lengths of a main canal and the secondary canals (Figure 1C); second, interim placement of an intracanal dressing of calcium hydroxide, and final irrigation using BioPure® MTAD® (Dentsply Tulsa Dental Specialist, Johnson City, TN, USA); third, using the tactile examination technique of #6 C+ files (Dentsply Maillefer, Tulsa, OK, USA) with pre-curved tips (13); and finally, augmentation of the flow dynamics during irrigation using the EndoActivator® (Dentsply Sirona, Santa Barbara, CA, USA).

During the tactile examination, the orifices of the secondary canals were not visible despite using the dental microscope because the orifices were located beneath the wall of the main canal (Figure 1D). Using the CBCT images, MB, L, DB, and ML canals were located in order. The glide pathways of the canals were achieved by the alternative use of #6 C+ files (Dentsply Maillefer, Tulsa, OK, USA) and S1 ProTaper Gold® (Dentsply Sirona, Johnson City, TN, USA). Later, the working lengths were measured as 24 mm in all canals. The canals were instrumented up to 25/.04 files in MB and ML canals, and 35/.04 rotary files in DB and L canals. The canals were irrigated using 1.3% sodium hypochlorite while negotiating and shaping the canals. Calcium hydroxide paste was placed in the canals and temporary sealing achieved using cotton pellets and Cavit. After a 1-month interval, the treatment was resumed. The MB, L, DB, and ML canals were cleaned using 35/.04 rotary files and a 1.3% sodium hypochlorite irrigant while removing calcium hydroxide. Three radiographs verifying the placement of the master cones were taken because the access was not wide enough for all master cones to be placed at once (Figure 1E). NSRCT was conducted following the advanced plan except for the distolingual canal (DL), which was not noticed until the obturation was completed (Figures 1F,G). The final irrigation was performed with sterilized water followed by the application of 20 ml of MTAD. The canals were obturated using EndoSequence BC sealer™ (Brasseler USA, Savannah, GA, USA) and an optimized technique using both single-cone and warm vertical compaction techniques.

### 2.2 Case 2

**Figure 2:**
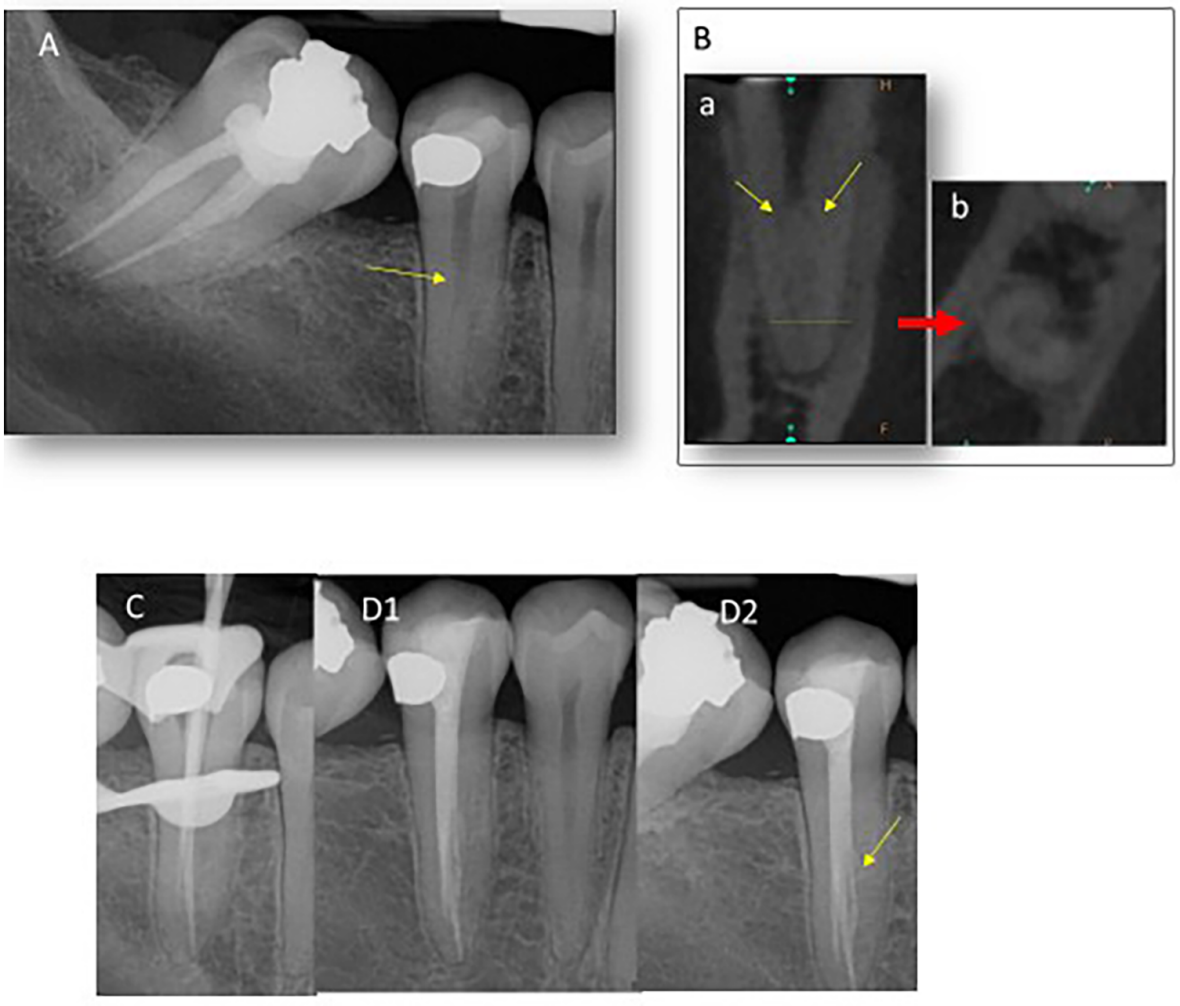
(**A**) A preoperative radiograph showing a fast break (yellow arrow) indicating the secondary canals exits. (**B**) A CBCT scan. (Left) A coronal view. Note that the secondary canals exit from a main canal at acute angles (yellow arrows). (Right) An axial view. Note the radicular groove mesially at the apical one-third of the root and it is obscure to count the numbers of the secondary canals. (**C**) A radiograph showing trial gutta-percha cones in MB and L. (**D1,D2**) Differently angled postoperative radiographs. Note that the C-shaped fin with a canal was obturated (yellow arrow).

A 32-year-old patient was referred for endodontic treatment of tooth 45. The diagnoses were symptomatic irreversible pulpitis and symptomatic apical periodontitis (Figure 2A). CBCT imaging was preoperatively employed because of the complicated root anatomy. The CBCT volume revealed one main canal and two or three secondary canals in addition to a radicular groove (Figure 2B). The advanced clinical strategy was planned and implemented: first, premeasuring the working lengths of the main canal and the secondary canals; second, intracanal dressing with calcium hydroxide; third, the tactile examination; and finally, augmentation of the flow dynamics using ultrasonic irrigation with an IrriSafe file (ACTEON, Mérignac, Aquitaine, France) for 20 s, three cycles in 25% power mode, and an EndoActivator® with 6% sodium hypochlorite.

After the access opening was completed, two secondary canals [buccal canal (B) and lingual canal (L)] and a C-shape configuration were detected (Figure 2B). The complicated canals were instrumented up to 25/.04 files and filled with a warm vertical compaction technique using two gutta-percha cones and AH Plus Jet sealer (Dentsply Maillefer, Tulsa, OK, USA) (Figures 2C,D).

## 3 Discussion

It is challenging to successfully complete NSRCT for a mandibular second premolar with multiple canals and a radicular groove (13). First, it is difficult to locate the orifices of the secondary canals because the secondary canals exit the main canal at the middle one-third of the root (Figures 1C, 2B-a). In addition, the secondary canals branch from the main canal at acute angles (Figures 1C, 2B-a) (14). Second, the secondary canals incorporate fins or a C-shape canal configuration in the characteristically elongated and thin root (Figures 1B, 2B-b) (12). These spaces are more difficult to access and disinfect using standard cleaning and shaping protocols. Third, there are two contradictory demands in play. One is to shape the canals thoroughly for disinfection. The other is to preserve tooth structure considering the thin dimensions of roots with a radicular groove. Therefore, it is necessary to limit the sizes and tapers of the files used to shape the canals. For these reasons, it is imperative to be aware of the anatomy of the canals and the cross-sectional dimensions of the root.

**Figure 3:**
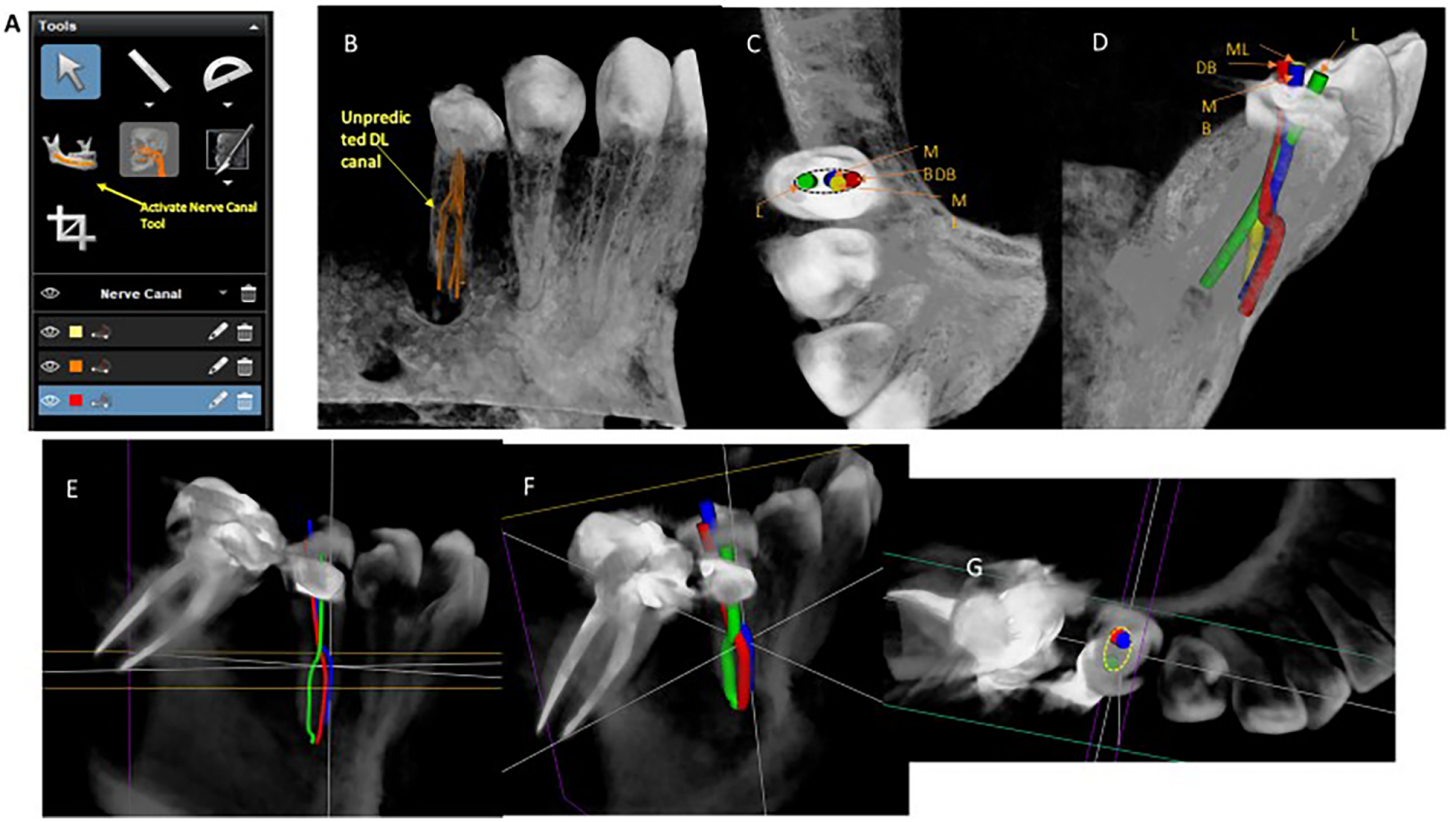
Simulation of the root canals and NSRCT. (**A**) Tools of CS 3D Imaging v3.8.7. Note the “Activate Nerve Canal Tool” which was retrospectively used for the simulation of NSRCTs in Case 1 and 2. (**B–D**) Case 1. Note 5 canals that are corresponding to root canal filling on the postoperative radiographs. (**B**) The diameter of the nerve by 500 μm was default in the “Activate Nerve Canal Tool”. (**C,D**) Simulating the outline of the access and the mechanical shaping in NSRCT. The diameters were changed from 500 μm to 1.5 mm. (**E–G**) Case 2. Note 3 secondary canals exit from a main canal at acute angles: green-L, red-DB and blue-MB. (**F,G**) Simulating the outline of the access and the mechanical shaping in NSRCT. The diameters of the tool were changed from 500 μm to 1.5 mm.

Conventional radiographs provided limited information about the configuration of the root canals (Figures 1A, 2A), while CBCT was useful in predicting the configuration of the root canals as well as the root dimensions. In case 1, despite using CBCT, a DL was not detected until the obturation was performed (Figures 1F,G). Similarly, only two canals were located in case 2, but it turned out there were three root canals with a C-shape in case 2 (Figure 2). Then, the authors ran retrospective simulations of these NSRCTs using the Nerve Canal tool in CS 3D Imaging v3.2.9 and v3.8.7 (Figure 3A) to trace and three-dimensionally visualize the canal systems’ configurations (Figures 3B–G). Changing the diameter and color modes of the tracing tool allowed for identifiable representation of the access outline and orientation of the secondary canals (Figures 3C,G).

The working lengths were pre-emptively obtained using CBCT scan images (Figure 1C) and the operators were able to premeasure at which level the secondary canals exited the main canals (Figure 1C). This was useful information when performing a tactile examination of the main canal walls to detect the secondary canals (14).

A DG-16 endodontic explorer is commonly used for the detection of the primary canal orifices during NSRCT. The previous case report recommended the use of small files with pre-curved tips to detect the orifices of the secondary canal branching off the main canal during NSRCT for mandibular premolars (14). By using this technique, considerable root structure can be preserved, which otherwise would be sacrificed during enlargement of the main canal (Figure 1D).

Mechanical instrumentation alone is not sufficient to completely debride the infected canal space within mandibular premolars with the standard single canal morphology (15). It is imperative to clinically incorporate chemo-mechanical cleaning and shaping methodologies during endodontic treatment. In these two reported cases, the presence of secondary canals with unpredictable fins or a C-shape configuration, which provide spaces for bacteria to regrow and form biofilms, necessitated using an intracanal dressing of calcium hydroxide for at least 7 days (11). The sonic irrigation EndoActivator System was also used for debridement in both cases. It has been reported that there is a lack of conclusive evidence to indicate superiority of the sonic irrigation method compared to static irrigation in *ex vivo* studies (16, 17). These studies contained only single-rooted teeth with a simple single canal. However, in other ex vivo studies, it was reported that the significant effectiveness of sonic irrigation in the curved roots of mandibular molars was demonstrated (18). In case 2, ultrasonic irrigation was used to augment debridement because it enhances the chemo-mechanical removal of necrotic tissues from complicated canal systems (19).

MTAD was used as a final rinse in case 1 as its use as an irrigant provides antimicrobial efficacy and removal of the smear layer by 3% doxycycline and 4.25% citric acid, respectively. Moreover, the doxycycline of MTAD has substantivity with dentine that suppresses reinfection by the residual bacteria in the untouched canal wall and/or dentinal tubules (20, 21). The efficacy of MTAD to remove the smear layer is augmented when a low concentration of sodium hypochlorite is used (22). A randomized clinical trial showed that using MTAD did not increase the healing rate in a single-visit treatment (23). However, single-rooted teeth (incisor, canine, and premolar) were tested in this clinical trial study.

In case 1, EndoSequence BC sealer was used in the optimized root canal filling technique. If the secondary canals were not observable or accessible beneath their orifices (Figures 1B–D), it is recommended to utilize the single-cone obturation technique for the secondary canals. This involves searing off the master cones and compacting them at the orifice level of the secondary canals. The main canal was then obturated using the warm vertical compaction technique. It has been reported that the success rates of initial NSRCT and non-surgical retreatment using the single-cone technique with EndoSequence BC sealer are 90.6% and 91.7%, respectively (23). The prognostic factor was only the size of an existing periradicular lesion, while the occurrence of extruded sealer did not negatively affect the outcomes (24).

## 4 Conclusions

The incidence of multiple root canals in mandibular second premolars with a radicular groove is extremely rare, and the configuration of the canals can be exceptionally complex and unpredictable. Therefore, it is critical to establish a precise and proper clinical strategy for treating such cases in order to achieve a successful clinical outcome.

## Data Availability

The original contributions presented in the study are included in the article/Supplementary Material, further inquiries can be directed to the corresponding author.
